# Intravenous thrombolysis for acute cerebral embolism during atrial fibrillation ablation: a case report and systematic review with meta-analysis

**DOI:** 10.3389/fcvm.2026.1801300

**Published:** 2026-04-17

**Authors:** Bin Cai, Peng Yuan, Li-Na Fan, Lin Ma, Tian-Tong Niu, Rong Bai

**Affiliations:** 1 Department of Neurology, Plastic Surgery Hospital, Chinese Academy of Medical Sciences, Beijing, China; 2 Department of Neurology, Beijing Anzhen Hospital, Capital Medical University, Beijing, China; 3 Department of Cardiology, Beijing Anzhen Hospital, Capital Medical University, Beijing, China

**Keywords:** dabigatran, idarucizumab, intravenous thrombolysis, ischemic stroke, radiofrequency ablation, systematic review

## Abstract

**Introduction:**

Periprocedural stroke represents a clinically important complication of atrial fibrillation ablation, and effective anticoagulation remains essential for risk reduction. However, the use of intravenous thrombolysis in patients with acute ischemic stroke who have recently received novel oral anticoagulants remains controversial under certain clinical circumstances. This report described an uncommon case of radiofrequency ablation-related ischemic stroke treated with intravenous thrombolysis after dabigatran reversal with idarucizumab. In parallel, we conducted a systematic review and meta-analysis to evaluate the safety and outcomes of this treatment strategy.

**Methods:**

This study included analysis of an index case and a systematic review with meta-analysis. We report a 54-year-old patient who developed acute ischemic stroke shortly after radiofrequency catheter ablation for AF and was treated with intravenous thrombolysis after dabigatran reversal with idarucizumab. In parallel, a systematic literature search was conducted to identify observational studies, cohorts, and case series reporting idarucizumab-enabled thrombolysis in dabigatran-treated stroke patients. Data on intracranial hemorrhage (ICH), favorable functional outcome [modified Rankin Scale (mRS) 0–2], and mortality were extracted. Pooled estimates were calculated using random-effects meta-analysis of proportions, and comparative analyses with non-anticoagulated thrombolysis populations were performed using pooled risk ratios.

**Results:**

Neuroimaging confirmed an acute cerebral infarction in the left thalamus. The patient had been receiving dabigatran and was treated with intravenous idarucizumab followed by intravenous alteplase. Neurological deficits rapidly improved after thrombolysis, and the patient achieved a favorable recovery over 90 days of follow-up.

**Discussion:**

Findings from the systematic review and meta-analysis demonstrated low rates of intracranial hemorrhage, low mortality, and favorable functional outcomes comparable to standard thrombolysis populations. This case, together with accumulating evidence from the literature, supports the short-term safety and clinical effectiveness of idarucizumab-enabled intravenous thrombolysis in dabigatran-treated patients with periprocedural acute ischemic stroke. Therefore, early anticoagulant reversal followed by prompt reperfusion therapy may represent a feasible and safe therapeutic strategy in carefully selected patients.

## Introduction

At present, most patients with non-valvular atrial fibrillation (AF) receive vitamin K antagonists or non–vitamin K oral anticoagulants for stroke prevention; however, a proportion of these patients still experience ischemic stroke or systemic thromboembolism (1). According to current guidelines for the early management of ischemic stroke, intravenous thrombolysis is contraindicated in patients with AF who are receiving novel oral anticoagulants (2). Recently, a new therapeutic strategy has been proposed for patients undergoing anticoagulation therapy. Specifically, intravenous thrombolysis may be considered after reversal with idarucizumab in patients treated with dabigatran, provided that anticoagulant parameters support its use (3, 4). Nevertheless, whether thrombolytic therapy can be safely performed under these circumstances remains controversial. Here, we report a case of successful intravenous thrombolysis in a Chinese patient with ischemic stroke who was receiving dabigatran following AF ablation, after anticoagulation reversal with idarucizumab. In addition, we present a literature review of published reports describing idarucizumab-enabled intravenous thrombolysis in dabigatran-treated patients with acute ischemic stroke.

## Methods

This systematic review and meta-analysis was conducted in accordance with the Preferred Reporting Items for Systematic Reviews and Meta-Analyses guidelines and followed established methodological standards for the synthesis of observational studies. The analysis focused exclusively on studies reporting outcomes in patients who underwent intravenous thrombolysis after dabigatran reversal with idarucizumab.

### Search strategy and study selection

We systematically screened the literature for cohort studies, observational studies, and case series reporting outcomes of idarucizumab-assisted intravenous thrombolysis in acute ischemic stroke. Studies were eligible if they reported at least one of the following outcomes of interest: (1) intracranial hemorrhage (ICH), (2) favorable functional outcome, defined as a modified Rankin Scale (mRS) score of 0–2, or (3) mortality. Duplicate datasets and reports without extractable quantitative data were excluded. During literature screening, studies reporting endovascular intervention after idarucizumab reversal were excluded. Because such reports were limited in number and the relevant procedural and outcome data were inconsistently reported across studies, meaningful quantitative synthesis was not feasible. Therefore, the present analysis focused exclusively on studies evaluating outcomes following intravenous thrombolysis after dabigatran reversal with idarucizumab.

### Data extraction

For each eligible study, we extracted study characteristics, sample size, total population, event counts, and outcome definitions as reported by the original authors. Extracted outcomes included ICH events, favorable functional outcome at follow-up, and all-cause mortality. When outcomes were reported as proportions, raw event counts were derived to enable reconstruction of effect estimates.

### Statistical analysis

To estimate pooled proportions of ICH, favorable functional outcome, and mortality, we performed a single-arm meta-analysis of proportions using a random-effects model based on the DerSimonian–Laird method. Proportions were transformed using the variance-stabilizing double-arcsine transformation before pooling and were back-transformed for presentation of the final results. Between-study heterogeneity was quantified using the I^2^ statistic. For comparative analyses between idarucizumab-treated patients and thrombolysis controls not pretreated with dabigatran, we calculated risk ratio (RR) with 95% confidence intervals (CIs) using inverse-variance random-effects models. Comparative effect estimates were derived only from included studies that reported a non-anticoagulated intravenous thrombolysis control group within the same cohort. Statistical heterogeneity was assessed using Cochran's *Q* test and the I^2^ statistic, with values of 50% and 75% indicating substantial and considerable heterogeneity, respectively. Sensitivity analyses excluding small case series were planned to evaluate the robustness of the findings, in line with previously published approaches.

All statistical analyses were conducted using the *meta* and *metafor* packages in R (version 4.3; R Software for Statistical Computing, Vienna, Austria).

## Case presentation

**Figure 1:**
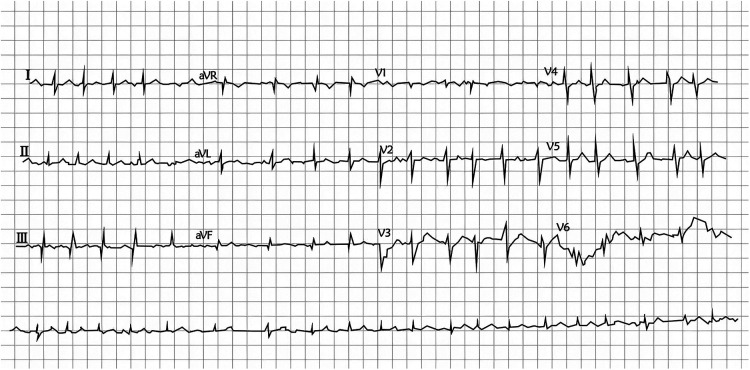
Twelve-lead electrocardiogram showing atrial fibrillation with a ventricular rate of 123 beats per minute and no significant ST–T changes.

A 54-year-old Han Chinese woman (height, 160 cm; weight, 51 kg) presented to the emergency department with worsening palpitations lasting 30 min, accompanied by dizziness and shortness of breath. During this episode, a 12-lead electrocardiogram demonstrated AF with a ventricular rate of 123 beats per minute and no significant ST–T changes (Figure 1). Cardiac enzymes, coagulation parameters, complete blood count, and renal and hepatic function tests were within normal limits (Table 1). Chest radiography revealed no abnormalities. Her symptoms improved after immediate treatment with sotalol, and she was subsequently admitted to the Department of Cardiology for further management.The patient reported an eight-year history of hypertension treated with allisartan isoproxil (240 mg once daily) for the preceding 8 months. She also reported suspected paroxysmal AF for 9 months without prior anticoagulant or antiarrhythmic therapy. Her personal and family histories were negative for diabetes, hyperlipidemia, and cardiovascular or cerebrovascular events. On admission, physical examination was unremarkable except for an irregular cardiac rhythm on auscultation and an irregular radial pulse of 90 beats per minute. Transesophageal echocardiography demonstrated no intracardiac thrombus.The patient had a CHA₂DS₂-VASc score of 1 and was started on dabigatran at a dose of 110 mg twice daily. Radiofrequency catheter ablation was performed 4 days after admission, primarily because of the rapid onset of anticoagulation with dabigatran and the low estimated risk of stroke or systemic embolism based on the CHA₂DS₂-VASc score. However, within 1 h after the procedure, she developed speech difficulty and right-sided weakness. Acute ischemic stroke was suspected, and a stroke neurologist was consulted.

**Table 1:** Renal and hepatic organ function.

Items	Measured value	Reference range
ALT (U/L)	9	7–40
AST (U/L)	32	13–35
*γ*-GT (U/L)	9	7–45
Urea (mmol/L)	2.26	2.6–7.5
Creatinine (*μ*mol/L)	47.1	41–81
Uric acid (μmol/L)	193.9	155–357

ALT, alanine aminotransferase; AST, aspartate aminotransferase; γ-GT, γ-glutamyl transferase.

**Figure 2:**
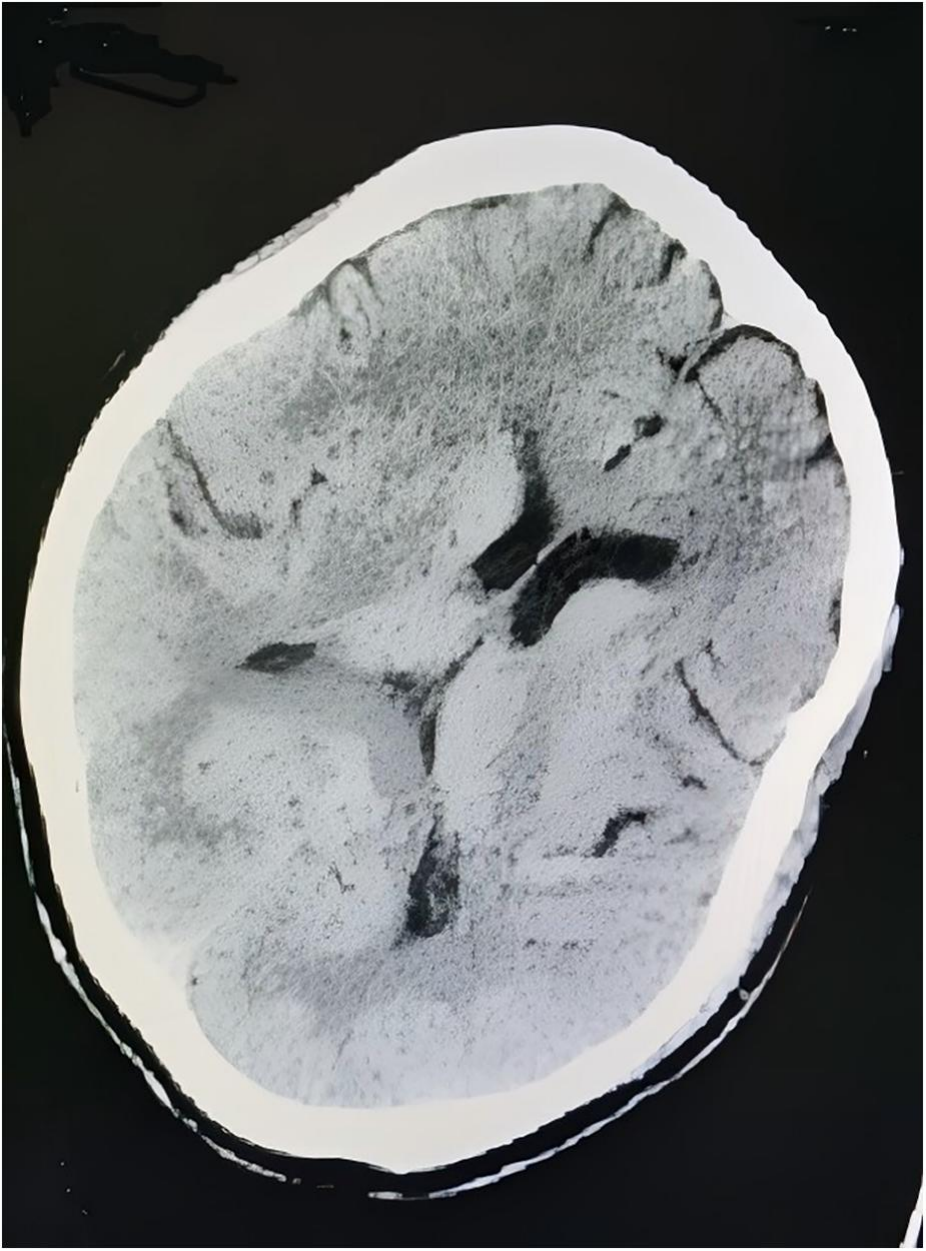
Urgent head computed tomography scan demonstrating no intracranial abnormalities 30 min after stroke onset.

**Figure 3:**
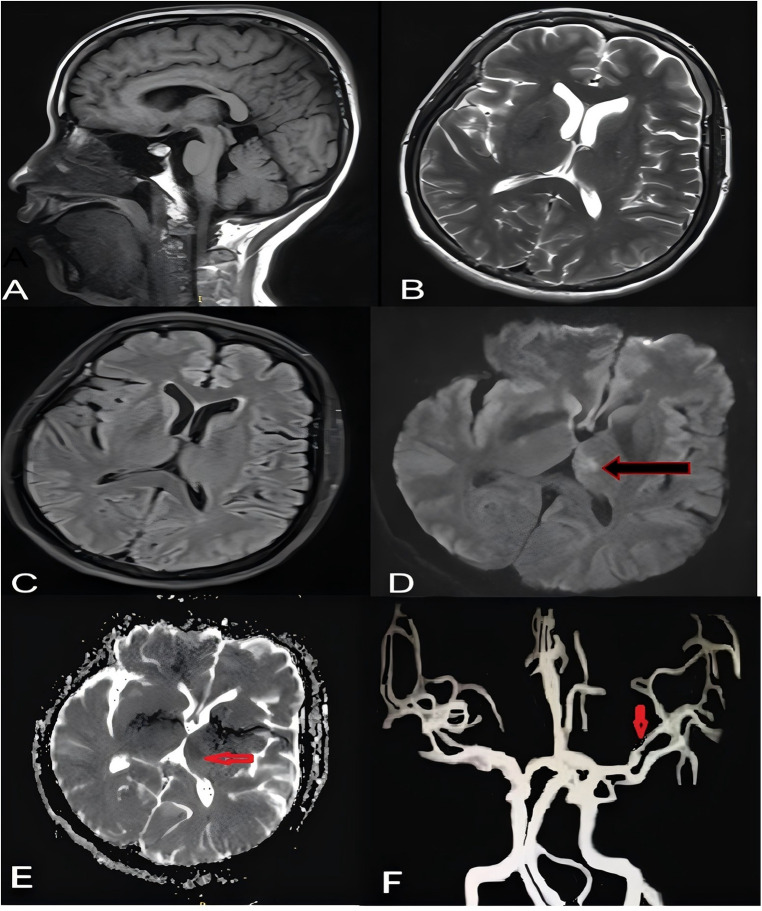
Brain magnetic resonance imaging performed 5 h after stroke onset. T1-weighted, T2-weighted, and fluid-attenuated inversion recovery images show no obvious abnormalities **(A–C)**. Diffusion-weighted imaging demonstrates a hyperintense lesion in the left thalamus [**(D)**, long black arrow] with corresponding hypointensity on the apparent diffusion coefficient map [**(E)**, short red arrow], consistent with acute infarction. Magnetic resonance angiography reveals mild stenosis of the superior trunk of the left middle cerebral artery [**(F)**, short red arrow].

**Figure 4:**
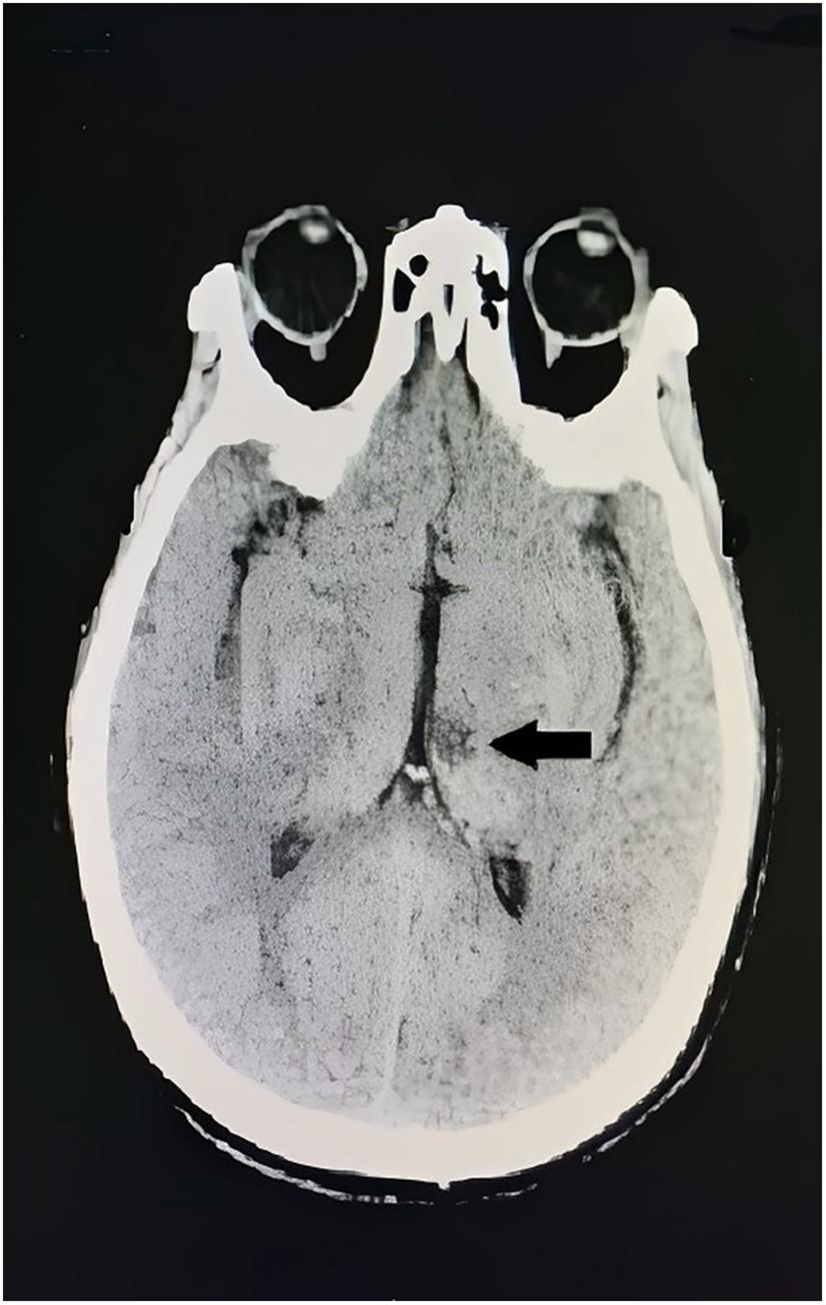
Follow-up head computed tomography scan performed 24 h after stroke onset showing a faint hypodense lesion in the left thalamus (black arrow).

**Figure 5:**
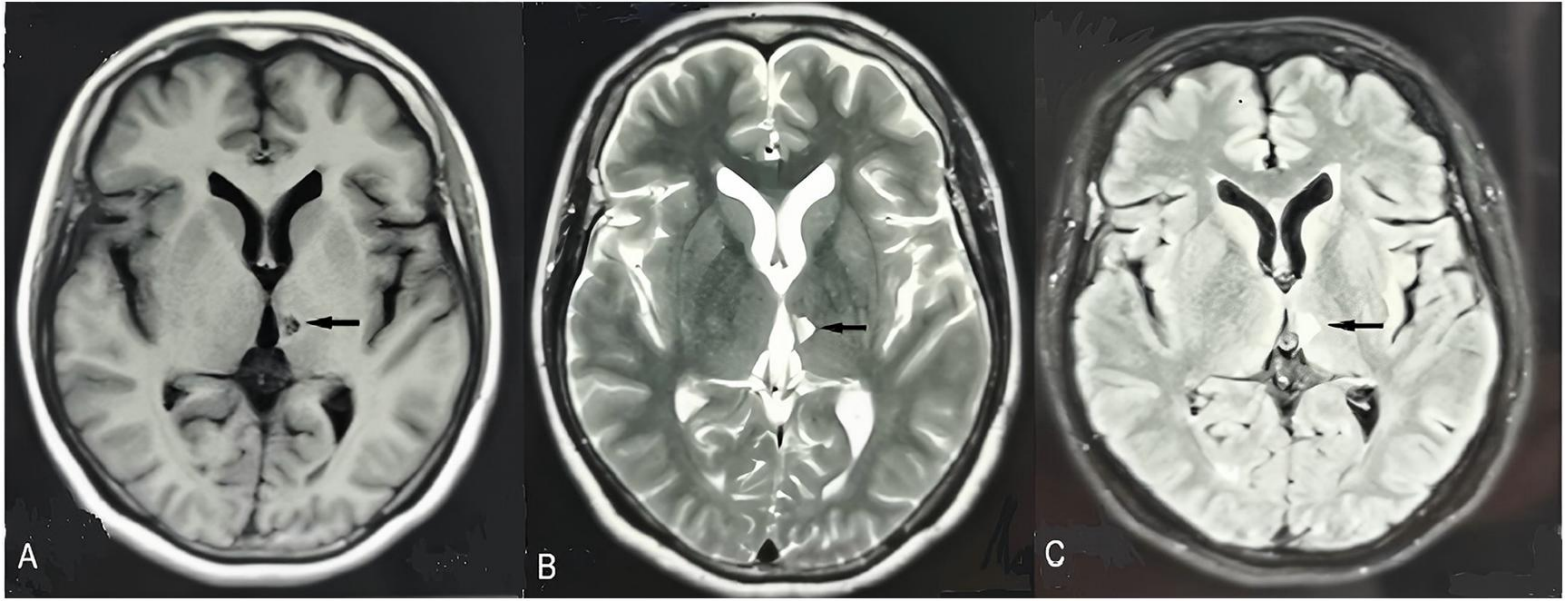
Brain magnetic resonance imaging obtained 3 months after hospital discharge. T1- weighted imaging shows hypointensity in the left thalamus **(A)**, with a small corresponding hyperintense lesion on T2-weighted and fluid-attenuated inversion recovery images [**(B)** and **(C)**, black arrow].

Neurological examination revealed lethargy, sensory aphasia, and complete paralysis of the right upper and lower limbs (muscle strength 0/5), with a National Institutes of Health Stroke Scale (NIHSS) score of 14. Vital signs recorded at that time were as follows: blood pressure 166/72 mmHg, heart rate 108 beats per minute, respiratory rate 22 breaths per minute, and body temperature 36.8 °C. An urgent head computed tomography scan performed 30 min after symptom onset showed no evidence of ICH (Figure 2). During the diagnostic workup, her neurological status deteriorated, progressing to superficial coma with rightward gaze palsy, and the NIHSS score increased to 20. After completion of neuroimaging and laboratory testing, including coagulation parameters (Table 2), cardiac enzymes, complete blood count, and a comprehensive metabolic panel, the neurologist promptly decided to proceed with thrombolytic therapy. Within 3 h of symptom onset, the patient received intravenous idarucizumab (5 g) administered over 15 min, followed by intravenous alteplase at a dose of 0.9 mg/kg body weight. Within 1 h after thrombolysis, her neurological deficits markedly improved, with residual right-sided hypoesthesia and mild hemiparesis. Muscle strength in the right upper and lower limbs improved to 5−/5, and the NIHSS score decreased from 20 to 1. The therapeutic timeline is summarized in Figure 3. Magnetic resonance imaging performed 5 h after stroke onset demonstrated a hyperintense lesion on diffusion-weighted imaging with a corresponding hypointense signal on the apparent diffusion coefficient map in the left thalamus, consistent with acute cerebral infarction. No abnormal signal changes were observed on T1-weighted, T2-weighted, or fluid-attenuated inversion recovery images (Figures 3A–E). Magnetic resonance angiography revealed mild stenosis of the superior trunk of the left middle cerebral artery (Figure 3F). On the following day, her neurological status remained stable, and a repeat head computed tomography scan showed a hypodense lesion in the left thalamus (Figure 4). Given the high risk of embolic events related to radiofrequency ablation and the low-to-moderate hemorrhagic risk indicated by neuroimaging findings and a HAS-BLED score of 2, anticoagulation with dabigatran (110 mg twice daily) was resumed 48 h after thrombolysis. The patient continued to recover steadily over the subsequent 7 days and was discharged on day 8 with only mild residual right-sided hypoesthesia and hemiparesis. At the 1-month follow-up conducted by telephone, the patient reported good adherence to her medications and no new symptoms. Transcranial Doppler ultrasonography subsequently confirmed normal cerebral blood flow in the bilateral middle cerebral arteries, right posterior cerebral artery, and bilateral vertebrobasilar arteries. At 3 months, her NIHSS score and a mRS score were both 0, indicating complete neurological recovery with no limitation in daily activities. A follow-up magnetic resonance imaging scan performed approximately 90 days after the initial study demonstrated a reduction in infarct size in the left thalamus (Figures 5A–C).

**Table 2:** Coagulation profile at different time points during treatment.

Time point	PT (s)	APTT (s)	INR
Before dabigatran	11.8	28.0	1.03
After dabigatran	14.4	65.1	1.27
After idarucizumab	14.2	53.5	1.25
1 h after thrombolysis	12.6	27.4	1.10
12 h after thrombolysis	13.1	27.1	1.14
Reference range	9.9–12.8	25.1–36.5	0.8–1.2

PT, prothrombin time; APTT, activated partial thromboplastin time; INR, international normalized ratio.

## Results

A total of 39 publications reporting approximately 480 individual cases of dabigatran-treated patients who underwent idarucizumab-enabled intravenous thrombolysis for acute ischemic stroke were identified. The included reports encompassed a wide range of study designs, including retrospective and prospective cohort studies, case series, and single-patient case reports, and demonstrated substantial heterogeneity in baseline characteristics, dabigatran dosing regimens, renal function, and the interval between the last anticoagulant dose and thrombolysis. Admission NIHSS scores varied widely, ranging from mild to severe neurological deficits. Coagulation profiles frequently demonstrated dabigatran-associated prolongation of activated partial thromboplastin time, thrombin time, or diluted thrombin time prior to reversal. Administration of idarucizumab resulted in rapid normalization of coagulation parameters, with intravenous thrombolysis typically initiated immediately or within minutes following reversal. Reported safety outcomes included ICH, mortality, and early neurological changes, while functional outcomes were most commonly assessed using the discharge NIHSS score or the mRS score at follow-up. Collectively, these cases provide a comprehensive clinical overview of idarucizumab-facilitated reperfusion therapy across diverse real-world settings (Table 3).

**Table 3:** Published cases identified in the literature.

Author (Year)	Study type	n	Age/Sex	Dabigatran regimen	Time since last dabigatran dose	Renal function (eGFR/Ccr)	NIHSS at admission	Coagulation tests on admission/post-antidote	Time from idarucizumab to thrombolysis	ICH	NIHSS at discharge	Functional outcome (mRS 0–2 = good)	Mortality (Deaths/Total)
Sanak et al. (2018) (25)	Retrospective series	13	70 ± 9.1; 7 M	150 mg bid	427 ± 235 min	48 ± 12 mL/s	medium 7 (3–23)	aPTT 38.1 ± 27.8 s, TT 72.2 ± 56.1 s/aPTT 29.5 ± 3.2 s, TT 16.5 ± 9.1 s	22 ± 18 min	3/13	3 (24 h)	10/13 good	0/13
Fang et al. (2019) (16)	Retrospective series	10	71 ± 8; 6 M	110/150 mg bid	9.7 ± 4.5 h	72 ± 17 mL/min	16.0 ± 6.67	aPTT 30.55 ± 5.72 s/ aPTT 26.82 ± 1.77s	11.11 ± 4.91 min	3/10	9.38 ± 4.75	NA	NA
Agosti et al. (2017) (14)	Case report	1	71 F	150 mg bid	NA	103 mL/min	9	aPTT 29 s (20–29.6)/aPTT 47 s	Immediate	No	NA	Good	0/1
Mutzenbach et al. (2016) (26)	Case report	1	68 M	110 mg bid	45 min	102 mL/min	3	aPTT 34 s, INR <1.2/NA	10 min	No	3	Good	0/1
Berrouschot et al. (2016) (27)	Case report	1	76 M	110 mg bid	3.5 h	normal	11	aPTT 73.3 s (20–40), TT 218 s (15–36)/aPTT 32.1 s, TT 17.2 s	Immediate	No	1	Good	0/1
Binet et al. (2018) (28)	Case report	1	55 F	150 mg bid	< 2 h	normal	20	aPTT 37.4 s (25.1–36.5), INR 1.18 (0.80–1.20), TT 80.7 (10.0–18.0) sec/aPTT 27.5 s, INR 1.14, TT 14.2 s	33 min	No	5	mRS=3	0/1
Xie et al. (2021) (29)	Case report	1	62 F	110 mg bid	24–48 h	68.6 mL/min	4	aPTT 32.7 s (28.00–42.50), PT 12.3 s (11.00–15.00), INR 1.06 (0.80–1.20)/APTT 29.4–31.9 s, PT 11.7–12.0 s, INR 1.01–1.05	35 min	No	1	Good	0/1
Lin et al. (2020) (20)	Case report	1	71 M	110 mg bid	<24 h	NA	9	INR 1.07/NA	10 min	No	2	mRS=3	0/1
Meyer et al. (2019) (17)	Case report	1	73 M	NA	<4 h	NA	19	INR of 1.1, PT of 12.5 s, and PTT of 27 s/PT 11.5–12.9 s, INR 1.1–1.2, and PTT 27–39 s.	15 min	No	1	Good	0/1
Kafke et al. (2016) (30)	Case report	1	75 F	110 mg bid	NA	NA	7	TT >150 s (14–21), PTT 35.5 s (23–36), INR1.10 (0.85–1.18)/NA	Immediate	No	18	NA	0/1
Kikule et al. (2022) (21)	Retrospective series	9	75.67 ± 8.59; 7 M	110/150 mg bid	NA	NA	9 (IQR=6.0–16.0)	NA	51 min (IQR=43–133)	1/9	4 (IQR=2.5–4.0)	4/9 good	NA
Jala et al. (2019) (31)	Case report	1	77 M	150 mg bid	3 h	normal	11	aPTT 51.8 s(24–36), INR 1.5, TT 17.5 s(15–19)/aPTT 32 s, INR 1.2, TT 21.0 s	15 min	No	0	Good	0/1
Hieber et al. (2019) (32)	Case report	1	81 M	150 mg bid	12 h	NA	6	TT 61.7 s/TT 18.3 s	50 min	No	4	NA	0/1
Pikija et al. (2017) (33)	Review+cases	21	76 (70–84)	110/150 mg bid	45min-17 h	NA	9.7 ± 6.17 (*n* = 20)	NA	NA	1/21	3.4 ± 5.50 (*n* = 18)	16/19 good	0/5
Amin et al. (2022) (34)	Case report	2	83 M/84 F	NA	3 h	NA	11/9	NA	Immediate/15min	No	9/4	1/2 good	1/2
Beharry et al. (2020) (35)	Retrospective series	13	79 (IQR 69–85); 9 M	NA	medium 149 min(*n* = 7)	NA	6 (IQR 4–21)	TCT median 80 s (IQR 57–113)/NA	NA	2/13	2(IQR 0.5–3.5)	8/13 good	2/13
Frol et al. (2020) (36)	Prospective series	19	75 ± 11.2; 10 M	110 mg bid	NA	NA	medium 9 (range 18)	NA	10–20 min	1/19	NIHSS ≥8 improvement in 84%	mRS medium 1 (range 6)	2/19
Kermer et al. (2020) (37)	Retrospective series	80	75.9 ± 10.7; 51 M	110/150 mg bid	NA	70.4 ± 19.6 mL/min	9.7 ± 5.1	aPTT 42.7 ± 15.1 s, TT 104.5 ± 63.9/NA	NA	No	Medium 2	mRS 4 → 2	3/80
Pretnar Oblak et al. (2019) (38)	Retrospective series	11	73.7 ± 12.5; 6 M	110/150 mg bid	NA	NA	Median 10	NA	10–20 min	2/11	Median 1	9/11 good	2/11
Vukorepa et al. (2020) (19)	Case report	1	80 F	110 mg bid	5 h 42 min	NA	8	NA/aPTT 24s(normal)	Immediate	No	1	good	0/1
Frol et al. (2021) (39)	Prospective series	22	75 ± 10.0; 11 M	NA	NA	NA	medium 10.5 (range 18)	NA	10–20 min	5/22	Mean NIHSS improvement 7.8 ± 3.5; improvement in 86% of patients	19/22 good	0/22
Alvarez Bravo et al. (2017) (40)	Case report	1	65 F	NA	14 h	NA	19	aPTT 31 s/NA	Immediate	No	1	Good	0/1
Baule et al. (2018) (41)	Case report	1	89 F	110 mg bid	NA	eGFR 38 mL/min	4	aPTT 38.80 s/NA	5 min	No	2（at 2 h)	Good	0/1
Barber et al. (2020) (42)	Observational cohort	51	Median 73 (57–83); 14 F	NA	NA	NA	8 (5–17)	NA	NA	2/51	NA [NIHSS change 3 (1.5–5.5)]	NA	2/51
Włodarczyk et al. (2023) (43)	Retrospective cohort	19	Median 74 (IQR 68–81); 13 M	110/150 mg bid	<12 h (68.4%), 12–24 h (26.3%), 24–48 h (5.3%)	NA	Median NIHSS 8 (IQR 6–12)	aPTT 35.1 s (IQR 29.2–40.8)/NA	median 63 min (IQR 47–86)	3/19	Median NIHSS 2 (IQR 0.75–3.25)	13/19 good	1/19
Gawehn et al. (2016) (44)	Case report	1	75 M	110 mg bid	∼9.5 h	NA	5	INR 1.01, aPTT 39.0 s, TT 66.8 s/normalized	Immediate	No	Symptoms resolved	Good	0/1
Hosoki et al. (2018) (45)	Case report	1	74 M	110 mg bid	∼4 h	Creatinine clearance 67.6 mL/min	6	aPTT 41 s/aPTT 27 s	Immediately	No	2	Good	0/1
Lo et al. (2018) (46)	Case report	1	78 F	110 mg bid	∼2 h	NA	34	APTT 50.7 s, TT 101.0 s, PT 16.8 s/aPTT 29.5 s, TT 17.4 s, PT 12.8s	10 min	No	Returned to baseline by day 7	Good outcome	0/1
Loh et al. (2019) (47)	Case report	1	77 M	110 mg bid	NA	eGFR 52 mL/min/1.73m^2^	8	INR 1.1, APTT 28.6 s, PT 14.0 s/NA	12 min	No	8 (Day 1)	NA	0/1
Ng et al. (2017) (48)	Case series	2	Case 1: 85 M; Case 2: 46 M	Both 110 mg bid	Case 1: 17 h; Case 2: 1 h	NA	Case 1: 30; Case 2: 5	Case 1: TT >60 s/NA; Case 2: NA	Immediate	2/2	Case 1: death Day 4; Case 2: NIHSS 18	0/2	1/2
Schäfer et al. (2016) (49)	Case report	1	67 F	150 mg bid	4 h	NA	10	TT 130 s/normalized	5 min	No	NA	0/1	0/1
Ohtani et al. (2019) (50)	Case report	1	67 F	Dabigatran 110 mg bid	NA	Not reported	7	aPTT 68.0 s/aPTT 43.2 s	5 min	No	NIHSS improved from 7 → 4 at 60 min	mRS 2 (good)	0/1
Ohya et al. (2018) (51)	Case report	1	57-year-old man	110 mg twice daily	NA	NA	22	aPTT 41.3 s on admission/NA	Immediately	No	Improved from 22 to 16 at 60 min; NIHSS 7 at day 30	mRS 3 at day 30	0/1
Romoli et al. (2022) (52)	Nationwide prospective–retrospective cohort	39	75.3 ± 7.4; 53.8% female	NA	404 min median	Not reported	Median 10 (IQR 8–15)	aPTT 34.8 ± 14.4 s, INR 1.2 ± 0.3/NA	NA	4/39	Not reported	25/39 good at 90 days	7/39
Schulz et al. (2016) (15)	Case report	1	76-year-old woman	110 mg bid	9 h	NA	11	TT 72.2 s/TT 20 s	Immediate	No	NIHSS 4 (2 h), 3 (24 h), 1 (7 days)	Good	0/1
Theodorou et al. (2024) (53)	Retrospective series	10	73.7; 5M	110/150 mg bid	NA	NA	12 ± 7	NA	Immediate	1/10	NA	5/10 good	2/10
Tsai et al. (2018) (54)	Case series	2	Case 1: 78 F; Case 2: 57 M	110 mg bid	Case 1: 16 h; Case 2: 9 h	eGFR 64.4/81.9	m Case 1: 24; Case 2: 9	Case1: aPTT 26.8 s/NA; Case2: aPTT 25.1 s/NA	NA	2/2	Case 1: death; Case 2: NIHSS 6 at 24 h	1/2	1/2
Tse et al. (2017) (55)	Case series	7	68 (52–78); 3F/4M	110 mg bid or 150 mg bid	NA	NA	Median 21 (6–28)	NA	Median 31 min	1/7	24-h NIHSS: variable (0–19)	2/7	0/7
Vosko et al. (2017) (56)	Case series	3	78 M/84 M/68 M	110–150 mg bid	NA	eGFR 68.8/49/Ccr 102 mL/min	9/9/3	aPTT 34–47 s, TT >150 s, dTT 34–134 ng/mL/normalized after antidote	20–25 min	0	Improved to NIHSS 0–4	Good	0/3

NIHSS, National Institutes of Health Stroke Scale; ICH, intracerebral hemorrhage; mRS, modified Rankin Scale; TT, thrombin time; TCT, thrombin clotting time; aPTT, activated partial thromboplastin time; PT, prothrombin time; INR, international normalized ratio; PTT, partial thromboplastin time; Ccr, creatinine clearance; eGFR, estimated glomerular filtration rate; IQR, interquartile range; NA, not available.

### Patient characteristics

Across published case reports and retrospective series, patients who received idarucizumab prior to intravenous thrombolysis were predominantly older adults, most commonly in seventh to ninth decades of life, with a slight male predominance reported in several cohorts. Baseline stroke severity was generally mild-to-moderate (NIHSS score ≤ 15). Renal function was typically preserved or only mildly impaired in cases where creatinine clearance was reported, indicating that severe renal dysfunction was uncommon among the included patients.

### Dabigatran exposure prior to thrombolysis

Most patients were treated with standard dabigatran dosing regimens of 110 mg or 150 mg twice daily. The interval between the last dabigatran dose and hospital presentation varied widely, ranging from <1 h to >12 h. In larger series, the interval from the last dabigatran dose to hospital presentation was typically several hours (reported as mean/median values), whereas case reports described both very short and prolonged intervals. Overall, no consistent exposure window was identified that was associated with poorer clinical outcomes.

### Coagulation profiles and reversal response

At presentation, most patients exhibited evidence of coagulation abnormalities, typically manifested as elevated activated partial thromboplastin time, thrombin time, or dabigatran-calibrated assay values, consistent with residual anticoagulant activity. Following administration of idarucizumab, available post-reversal laboratory data consistently demonstrated rapid normalization of coagulation parameters, often within minutes. Although some retrospective cohorts did not report post-reversal laboratory values, the overall evidence supports a reliable and immediate reversal of the anticoagulant effect of dabigatran.

### Interval between idarucizumab administration and intravenous thrombolysis

In nearly all reports, intravenous thrombolysis was initiated shortly after idarucizumab infusion, most commonly within 10–30 min and, in several cases, immediately. This short interval reflects a streamlined clinical workflow and supports the practical feasibility of promptly administering thrombolytic therapy after pharmacologic reversal. Larger cohorts that did not report precise timing nonetheless described a similar procedural approach.

### Safety profile and hemorrhagic complications

Across studies, the incidence of hemorrhagic complications following reversal-enabled thrombolysis was low. Although small series occasionally reported hemorrhagic transformation or symptomatic ICH, the absolute event numbers were limited and generally comparable to those observed in standard intravenous thrombolysis populations. ICH rates in standard intravenous alteplase-treated populations vary by definition and cohort, including 6.4% in the National Institute of Neurological Disorders and Stroke (NINDS) rt-PA Stroke Trial, 2.4% in the European Cooperative Acute Stroke Study III (ECASS III) trial, and 1.7% in the Safe Implementation of Treatments in Stroke–Monitoring Study (SITS-MOST) registry (5, 6). Most individual case reports documented no post-treatment bleeding events. Overall, the available evidence suggests that idarucizumab-facilitated intravenous thrombolysis is not associated with a clear increase in hemorrhagic risk. Reporting of thromboembolic/ischemic events after idarucizumab reversal was inconsistent across the included publications. Most case reports or series did not provide systematic ascertainment or a defined follow-up window for embolic/ischemic complications. Due to substantial missingness and heterogeneity in outcome definitions and follow-up duration, we did not perform a quantitative synthesis for these events.

### Functional outcomes

Functional outcomes were favorable in the majority of reported cases. Most studies described substantial improvement in NIHSS scores during hospitalization and a high proportion of patients achieving favorable functional outcomes (mRS score, 0–2) at discharge or follow-up. Several case reports documented complete or near-complete neurological recovery, highlighting the potential clinical benefit of timely intravenous thrombolysis following effective dabigatran reversal.

### Meta-analysis

**Figure 6:**
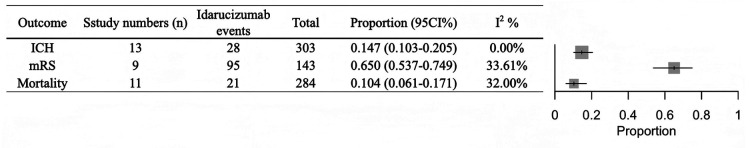
Forest plot presenting pooled proportions of ICH, favorable functional outcome (mRS score, 0–2), and mortality in patients with acute ischemic stroke treated with intravenous thrombolysis following dabigatran reversal with idarucizumab. ICH, intracranial hemorrhage; mRS, modified Rankin Scale; CI, confidence interval.

A total of 13 studies reported the incidence of ICH following intravenous thrombolysis after dabigatran reversal with idarucizumab. The pooled proportion of ICH was 0.147 (95% CI, 0.103–0.205), with no observed heterogeneity (I^2^ = 0%). Nine studies reported favorable functional outcomes, with a pooled proportion of patients achieving a mRS score of 0–2 of 0.650 (95% CI, 0.537–0.749) and moderate heterogeneity (I^2^ = 33.61%). Eleven studies reported mortality, yielding a pooled mortality proportion of 0.104 (95% CI, 0.061–0.171; I^2^ = 32.00%; Figure 6).

**Figure 7:**
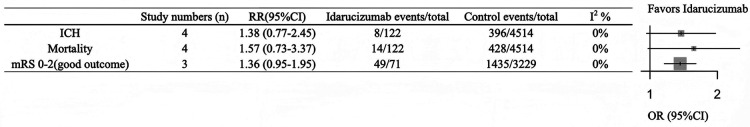
Forest plot summarizing random-effects pooled RRs for ICH, mortality, and favorable functional outcome (mRS score, 0–2) after intravenous thrombolysis following dabigatran reversal with idarucizumab. ICH, intracranial hemorrhage; mRS, modified Rankin Scale; OR, odds ratio; RR, risk ratio; CI, confidence interval.

Four comparative studies were available for analyses of ICH and mortality, and three for favorable functional outcomes. Compared with standard intravenous thrombolysis in non-anticoagulated control populations, idarucizumab-assisted thrombolysis was not associated with an increased risk of ICH (RR, 1.38; 95% CI, 0.77–2.45; I^2^ = 0%) or mortality (RR, 1.57; 95% CI, 0.73–3.37; I^2^ = 0%). Similarly, the likelihood of achieving a favorable functional outcome did not differ significantly between patients receiving idarucizumab-assisted thrombolysis and controls (RR, 1.36; 95% CI, 0.95–1.95; I^2^ = 0%; Figure 7). Overall, these findings suggest that idarucizumab-enabled intravenous thrombolysis yields functional outcomes comparable to standard thrombolysis, without a clear increase in hemorrhagic risk or mortality, although the limited number of comparative studies warrants cautious interpretation.

## Discussion

Catheter ablation has become a widely accepted and generally safe strategy for restoring sinus rhythm in patients with AF, particularly in those who do not respond to antiarrhythmic drug therapy. However, periprocedural stroke remains a clinically important complication, as thromboembolic events tend to cluster within the first 24 h after AF ablation and may persist for up to 2 weeks following the procedure (7). To reduce this risk, current guidelines recommend at least 3 weeks of effective anticoagulation with either warfarin or a novel oral anticoagulant before ablation, along with transesophageal echocardiography to exclude left atrial thrombus (8). In the present case, the periprocedural stroke likely occurred in the context of insufficient preprocedural anticoagulation of <3 weeks. The precise mechanism of the thromboembolic event remains uncertain; however, small thrombotic fragments, tissue debris, or air emboli rather than preformed cardiac thrombi may have contributed, given the small infarct size observed on magnetic resonance imaging and the absence of intracardiac thrombus on transesophageal echocardiography (7). Further evidence is needed to clarify these mechanisms.

Dabigatran, a novel oral anticoagulant, is widely used for the prevention of ischemic stroke and systemic embolism in patients with non-valvular AF (9, 10). Idarucizumab is a humanized monoclonal antibody fragment that specifically and effectively neutralizes dabigatran by irreversibly binding to it and abolishing its anticoagulant activity. Reversal rapidly occurs and persists for approximately 12 h (11), making idarucizumab suitable for use in life-threatening bleeding or situations requiring urgent intervention (12, 13). To date, a limited number of reports have described intravenous thrombolysis for ischemic stroke following dabigatran reversal with idarucizumab under various clinical circumstances (14–22). In the present case of radiofrequency ablation–related ischemic stroke, this therapeutic approach was selected based on three considerations: a definitive diagnosis of ischemic stroke; a short duration of dabigatran exposure prior to symptom onset; and the absence of an indication for neurointerventional therapy. As expected, the patient experienced rapid neurological improvement and a favorable short-term outcome, supporting the potential effectiveness of this strategy in carefully selected patients with recent dabigatran use.

Although idarucizumab itself has no intrinsic procoagulant properties, patients treated with dabigatran often have underlying thrombotic risk factors. Thrombotic events may therefore occur after reversal, particularly if anticoagulation is not promptly resumed. Raco et al. reported thrombotic complications, including deep venous thrombosis, pulmonary embolism, left atrial thrombosis, non–ST-segment elevation myocardial infarction, and ischemic stroke, following urgent idarucizumab administration, likely related to factors, such as advanced age, prior stroke, and higher CHA₂DS₂-VASc scores (14, 23). In the present case, the patient was middle-aged, had no history of stroke, and, importantly, anticoagulation was resumed 24 h after thrombolysis. Collectively, these factors likely contributed to the favorable short-term clinical outcome without severe adverse events.

Based on the present meta-analysis, idarucizumab-enabled intravenous thrombolysis in dabigatran-treated patients was associated with favorable functional outcomes comparable to those observed in standard thrombolysis populations. In standard intravenous alteplase-treated cohorts, functional good (mRS 0–2 at 3 months) has been reported at 54.8% in the Safe Implementation of Treatments in Stroke–International Stroke Thrombolysis Register datasets (24). Therefore, the observed outcomes in our synthesis appear to fall within the range reported in these standard thrombolysis populations. Specifically, the pooled proportion of patients achieving good functional recovery (mRS score, 0–2) reached approximately 65%, and comparative analyses demonstrated no statistically significant reduction in the likelihood of favorable outcomes compared with non-anticoagulated controls as shown in meta-analysis. Although the pooled relative risk for good functional outcome showed a numerically higher point estimate, the CI crossed unity, indicating comparable efficacy rather than superiority. These findings are consistent with previous observational cohorts and registry-based studies, which reported substantial neurological improvement and acceptable functional independence after timely thrombolysis following dabigatran reversal (5, 6, 18, 22). The available evidence supports the concept that rapid neutralization of dabigatran with idarucizumab can restore the effectiveness of intravenous thrombolysis and allow eligible patients to derive clinical benefit without compromising functional recovery.

Safety remains a central concern when considering thrombolysis in anticoagulated patients. The pooled incidence of ICH after idarucizumab-assisted thrombolysis was low and showed no significant increase compared with conventional thrombolysis. Importantly, comparative meta-analytic results demonstrated no statistically significant elevation in the risk of ICH or mortality relative to control populations not receiving dabigatran as shown in Figure 7. These findings are concordant with those of prior real-world studies and national registry data, which have consistently reported acceptable hemorrhagic complication rates following dabigatran reversal (8,25,28,38). Current evidence suggests that if appropriate patient selection and imaging-based exclusion criteria are applied, idarucizumab does not confer additional bleeding risk when used to facilitate thrombolysis.

This study has several strengths. It represents a comprehensive meta-analysis focusing specifically on the efficacy and safety of idarucizumab-enabled intravenous thrombolysis in dabigatran-treated patients, integrating data from diverse real-world settings and including both functional and safety endpoints. Nevertheless, several limitations must be acknowledged. Most included studies were observational in design, with inherent risks of selection bias and residual confounding. Sample sizes in individual studies were relatively small, and heterogeneity existed with respect to dabigatran dosing, timing of the last dose, renal function, and follow-up duration. In addition, the number of comparative studies remains limited, which restricts the statistical power to detect small differences in rare adverse outcomes. Another limitation is the inability to evaluate door to needle time and thromboembolic/ischemic complications after reversal and thrombolysis. Although the available evidence suggests acceptable short-term hemorrhagic safety of intravenous thrombolysis after idarucizumab reversal, conclusions regarding post-reversal thromboembolic risk should be interpreted cautiously.

Despite these limitations, the current findings have important practical implications. For patients with AF receiving long-term dabigatran therapy who present with suspected acute ischemic stroke, rapid clinical assessment and immediate non-contrast head computed tomography should be performed to exclude ICH. In the absence of contraindications to thrombolysis, prompt administration of a full dose of idarucizumab should be considered, followed by immediate intravenous thrombolysis without unnecessary delay for coagulation test results. Importantly, optimization of reversal-to-thrombolysis should be embedded within the broader acute reperfusion decision pathway. In dabigatran-treated patients presenting with suspected acute ischemic stroke, early assessment for large vessel occlusion via computed tomography angiography or magnetic resonance angiography remains critical to determine mechanical thrombectomy eligibility. When large vessel occlusion is identified and the patient meets criteria, mechanical thrombectomy should be considered while efforts continue to minimize treatment delays. Such a streamlined approach may help minimize time to reperfusion and optimize neurological outcomes. Further prospective studies and registry-based analyses are warranted to refine patient selection criteria, standardize treatment protocols, and better define long-term outcomes in this clinically challenging population.

## Conclusion

This unusual case of stroke related to post-ablation embolism supports the view that, in patients experiencing periprocedural ischemic stroke while receiving dabigatran, pretreatment with idarucizumab may provide short-term safety and effectiveness for intravenous thrombolysis. Nevertheless, additional clinical evidence remains necessary to clarify optimal screening and selection criteria for patients with radiofrequency ablation-related ischemic stroke, determine dosing strategies that maximize therapeutic efficacy, and minimize the risk of adverse events.

## Data Availability

The datasets presented in this article are not readily available because of ethical and privacy restrictions. Requests to access the datasets should be directed to the corresponding author/s.
